# Sintilimab plus anlotinib in later-line treatment of advanced KRAS-mutant NSCLC: a multicenter, retrospective case series

**DOI:** 10.3389/fmed.2026.1795432

**Published:** 2026-03-02

**Authors:** Yongjin Su, Chenyang Wang, Liyue Sun, Feng Jin, Xiangyu Wang, Shubin Wang, Fen Wang

**Affiliations:** 1 Department of ENT, Peking University Shenzhen Hospital, Shenzhen, Guangdong, China; 2 Department of Medical Oncology, Peking University Shenzhen Hospital, Shenzhen, Guangdong, China; 3 Health Management Center, The First Affiliated Hospital of Jinan University, Guangzhou, Guangdong, China; 4 Proof of Concept Center, The First Affiliated Hospital of Jinan University, Guangzhou, Guangdong, China

**Keywords:** anlotinib, anti-angiogenic therapy, case series, immunotherapy, *KRAS* mutation, non-small cell lung cancer, sintilimab

## Abstract

**Background:**

Patients with advanced *KRAS*-mutant non-small cell lung cancer (NSCLC) face a paucity of effective later-line therapies. While combining PD-1 inhibitors with anti-angiogenic agents is a promising strategy, real-world evidence for chemo-free combinations in this specific population remains scarce.

**Methods:**

This multicenter, retrospective case series evaluated 27 patients with advanced *KRAS*-mutant NSCLC who received sintilimab (200 mg IV, q3w) plus anlotinib (8–12 mg PO, d1–14, q3w) after ≥1 prior line of therapy. The primary endpoints were progression-free survival (PFS) and overall survival (OS). Secondary endpoints included objective response rate (ORR) and safety.

**Results:**

With a median follow-up of 30.3 months, the median PFS was 7.96 months (95% CI: 5.6–10.3) and median OS was 12.4 months (95% CI: 8.2–16.6). The ORR was 33.3% and the disease control rate was 92.6%. A critical finding was that patients without prior exposure to anti-angiogenic therapy had significantly superior PFS (9.1 vs. 3.0 months, HR = 0.29, *p* < 0.001) and OS (13.5 vs. 5.7 months, HR = 0.42, *p* = 0.025). Grade 3–4 treatment-related adverse events occurred in 25.9% of patients, primarily hypertension (11.1%) and hand-foot syndrome (7.4%), with no fatal events.

**Conclusion:**

This real-world case series suggests that sintilimab plus anlotinib offers promising efficacy and manageable toxicity as a later-line, chemotherapy-free regimen for advanced *KRAS*-mutant NSCLC. The absence of prior anti-angiogenic therapy emerged as a strong positive predictor for survival, underscoring the importance of strategic treatment sequencing in clinical practice.

## Introduction

*KRAS* mutations, occurring in 20–30% of non-small cell lung cancer (NSCLC) cases in Western populations and approximately 13% in Chinese patients, define a distinct molecular subset historically associated with limited therapeutic options and poor prognosis (1). Unlike *EGFR* or *ALK*-driven tumors, *KRAS*-mutant NSCLCs have historically been less amenable to targeted therapies. While immunotherapy (with or without chemotherapy) is now standard in the first-line setting, and KRAS G12C inhibitors (e.g., sotorasib, adagrasib) are approved in the second-line and beyond, access to these agents remains limited in many regions, including China, and efficacy in non-G12C subtypes is modest (2, 3).

Combining ICIs with anti-angiogenic agents represents a rational strategy to remodel the immunosuppressive tumor microenvironment (TME). Preclinically, anti-angiogenic drugs can normalize aberrant vasculature, enhance immune cell infiltration, and potentiate ICI activity (4). Clinically, regimens such as atezolizumab plus bevacizumab and chemotherapy have shown benefit in *KRAS*-mutant NSCLC, with a median PFS improvement of approximately 2 months in exploratory subgroup analyses (5). Notably, KRAS-mutant tumors are often characterized by an immunosuppressive tumor microenvironment, which may render them particularly susceptible to combined PD-1/PD-L1 and VEGF pathway blockade (6, 7). This provides a strong biological rationale for evaluating such combinations specifically in this molecular subset. However, chemotherapy-free combinations, particularly using oral multi-target tyrosine kinase inhibitors (TKIs) with ICIs, are underexplored in this molecular context within real-world Chinese practice.

Sintilimab, a fully human anti-PD-1 antibody, and anlotinib, a multi-target TKI against VEGFR, FGFR, and PDGFR, have demonstrated synergistic activity in advanced NSCLC (8). While a phase II trial reported encouraging first-line activity of this combination in driver-negative NSCLC (9), data specifically focusing on its efficacy and safety in pretreated *KRAS*-mutant patients are lacking.

Therefore, this multicenter, retrospective case series aimed to evaluate the real-world performance of sintilimab plus anlotinib in Chinese patients with advanced *KRAS*-mutant NSCLC who had progressed on prior therapies. Additionally, we sought to explore potential clinical predictors of benefit, with a specific focus on the impact of prior anti-angiogenic therapy, to inform optimal treatment sequencing.

## Methods

### Study design and participants

This multicenter, retrospective case series enrolled patients with histologically confirmed advanced KRAS-mutant NSCLC from three tertiary hospitals in the Guangdong-Shenzhen region between April 2019 and June 2022. The final follow-up date was March 4, 2024. Key inclusion criteria were: (1) age ≥18 years; (2) stage IIIB–IV disease (AJCC 8th edition) with a documented KRAS mutation detected by next-generation sequencing (NGS) of tumor tissue or plasma circulating tumor DNA (ctDNA); (3) disease progression after ≥1 prior line of systemic therapy; (4) completion of at least two cycles of sintilimab plus anlotinib; and (5) availability of complete radiological and safety data. Patients were excluded if they had mixed small-cell lung cancer histology, active autoimmune diseases requiring systemic immunosuppression, or a concurrent malignancy within the past 5 years. Data were collected using a standardized case report form across all participating centers. To ensure data quality and consistency, all data entries were reviewed by two independent investigators (FW and CW), with any discrepancies resolved through consensus or consultation with the principal investigator (YS). Site-specific data were pooled for analysis; due to the small sample size, center effect was not formally assessed but is acknowledged as a potential limitation. The study was approved by the institutional ethics committees of all participating centers (Approval No. SZYY-2023ER087), with a waiver of informed consent granted for the use of retrospectively collected, anonymized data.

### Treatment and assessments

Patients received sintilimab (200 mg) intravenously on day 1 of each 21-day cycle, and anlotinib orally once daily at a starting dose of 8–12 mg on days 1–14 of each cycle. The dose of anlotinib was adjusted at the physician’s discretion based on the patient’s Eastern Cooperative Oncology Group (ECOG) performance status and comorbidities. Treatment continued until disease progression (as per RECIST 1.1), unacceptable toxicity, or patient withdrawal. Dose reductions of anlotinib (to 8 mg daily) were permitted for managing grade 3 adverse events such as hypertension.

### Outcomes

The primary endpoints were progression-free survival (PFS), defined as the time from treatment initiation to radiological progression or death from any cause, and overall survival (OS), defined as the time from treatment initiation to death from any cause. Secondary endpoints included objective response rate (ORR) and disease control rate (DCR), both assessed by independent radiologists according to RECIST 1.1. Tumor assessments were conducted every two cycles. Treatment-related adverse events (TRAEs) were recorded and graded according to the National Cancer Institute’s Common Terminology Criteria for Adverse Events (CTCAE), version 5.0. Exploratory analyses evaluated baseline inflammatory and nutritional indices, including: Neutrophil-to-lymphocyte ratio (NLR): Calculated as absolute neutrophil count divided by absolute lymphocyte count from peripheral blood samples collected within 7 days prior to treatment initiation. Platelet-to-lymphocyte ratio (PLR): Calculated as absolute platelet count divided by absolute lymphocyte count from the same blood sample. Prognostic nutritional index (PNI): Calculated as 10 × serum albumin (g/dL) + 0.005 × total lymphocyte count (per mm^3^) from the same blood sample. Cutoff values for NLR (2.99) and PLR (176.33) were determined using median split, as no established cutoffs exist for this specific population. All indices were assessed at a single baseline time point prior to the first dose; therefore, dynamic changes during treatment were not evaluated.

### Statistical analysis

Categorical variables are summarized as frequencies and percentages, and continuous variables as medians with ranges. Survival curves were generated using the Kaplan–Meier method and compared with the log-rank test. Univariate Cox proportional hazards models were used to explore potential prognostic factors, including age, sex, ECOG performance status, KRAS G12C mutation status, and prior exposure to anti-angiogenic therapy or ICIs. Multivariate analysis was not performed due to the limited sample size and number of events, which would preclude stable model estimation; this limitation is addressed in the Discussion. All statistical tests were two-sided, with a *p*-value <0.05 considered statistically significant. Analyses were performed using SPSS Statistics (version 25.0) and GraphPad Prism (version 9.0). Given the exploratory, real-world nature of this study, no formal sample size calculation was performed; all eligible patients identified during the study period were included.

## Results

### Patient characteristics

A total of 27 patients with advanced KRAS-mutant NSCLC were included in this retrospective analysis. The median age was 63 years (range: 36–80), and most patients were male (85.2%, 23/27). Nearly all patients (96.3%) had adenocarcinoma histology, with one case of squamous cell carcinoma (3.7%). The most frequent KRAS subtypes were G12C (48.1%, 13/27), G12V (25.9%, 7/27), and G12D (18.5%, 5/27). Common sites of metastasis included the liver (14.8%), bone (18.5%), and brain (7.4%). The majority of patients had received prior chemotherapy (88.9%) or immune checkpoint inhibitors (66.7%), while a subset (22.2%, 6/27) had been exposed to anti-angiogenic agents before enrollment (Table 1). Of note, while 66.7% of patients had received prior immunotherapy (reflecting real-world practice and drug accessibility in China during the study period), all patients had documented disease progression on or intolerance to their most recent therapy prior to initiating the study regimen.

**Table 1:** Baseline clinical and pathological characteristics (*n* = 27).

Characteristic	Value
Median age, years (range)	63 (36–80)
Male sex, *n* (%)	23 (85.2%)
ECOG PS 0–1, *n* (%)	23 (85.2%)
Histology, *n* (%)	
Adenocarcinoma	26 (96.3)
Squamous cell carcinoma	1 (3.7)
KRAS subtype	
G12C	13 (48.1%)
G12V	7 (25.9%)
G12D	5 (18.5%)
Other	2 (7.4)
Brain metastases	2 (7.4%)
Prior anti-angiogenic therapy	6 (22.2%)
Prior lines of therapy, *n* (%)	
1 prior line	8 (29.6)
2 prior lines	12 (44.4)
≥3 prior lines	7 (25.9)

Data are presented as *n* (%) unless otherwise specified. ECOG PS, Eastern Cooperative Oncology Group Performance Status.

### Efficacy outcomes

**Figure 1:**
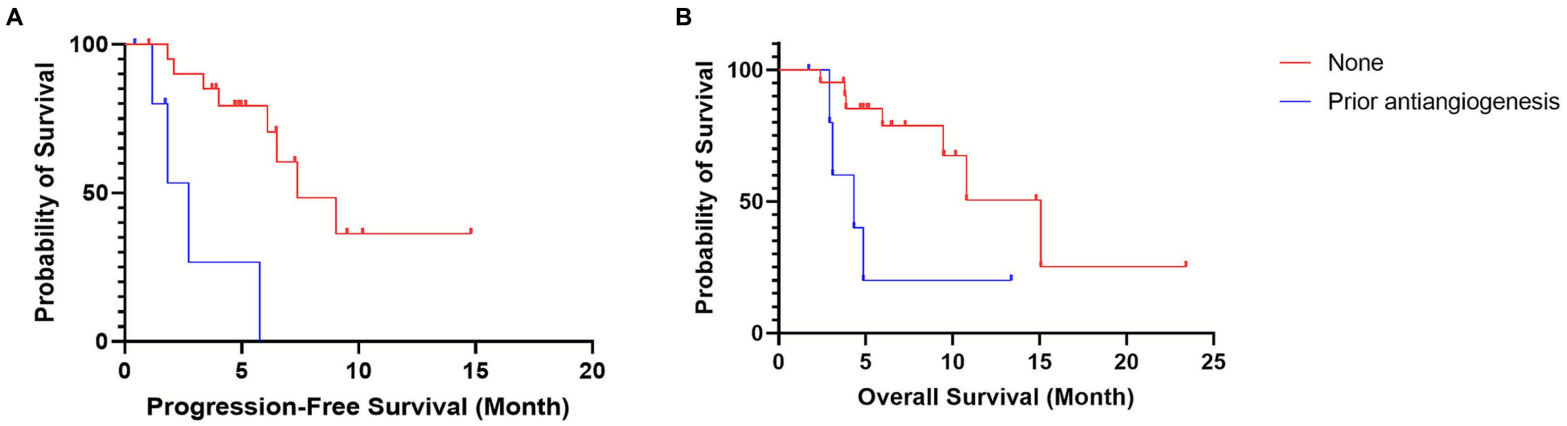
Kaplan–Meier curves for efficacy in the overall cohort. **(A)** Progression-free survival (PFS). **(B)** Overall survival (OS). Median PFS was 7.96 months (95% CI: 5.6–10.3). Median OS was 12.4 months (95% CI: 8.2–16.6). Tick marks represent censored observations.

After a median follow-up of 30.3 months, the combination of sintilimab and anlotinib demonstrated clinically meaningful antitumor activity. The median progression-free survival (PFS) was 8.0 months (95% CI: 5.6–10.3; Figure 1A), and the median overall survival (OS) was 12.4 months (95% CI: 8.2–16.6; Figure 1B). The objective response rate (ORR) was 33.3% (9/27), all of which were partial responses, and the disease control rate (DCR) reached 92.6% (25/27).

**Figure 2:**
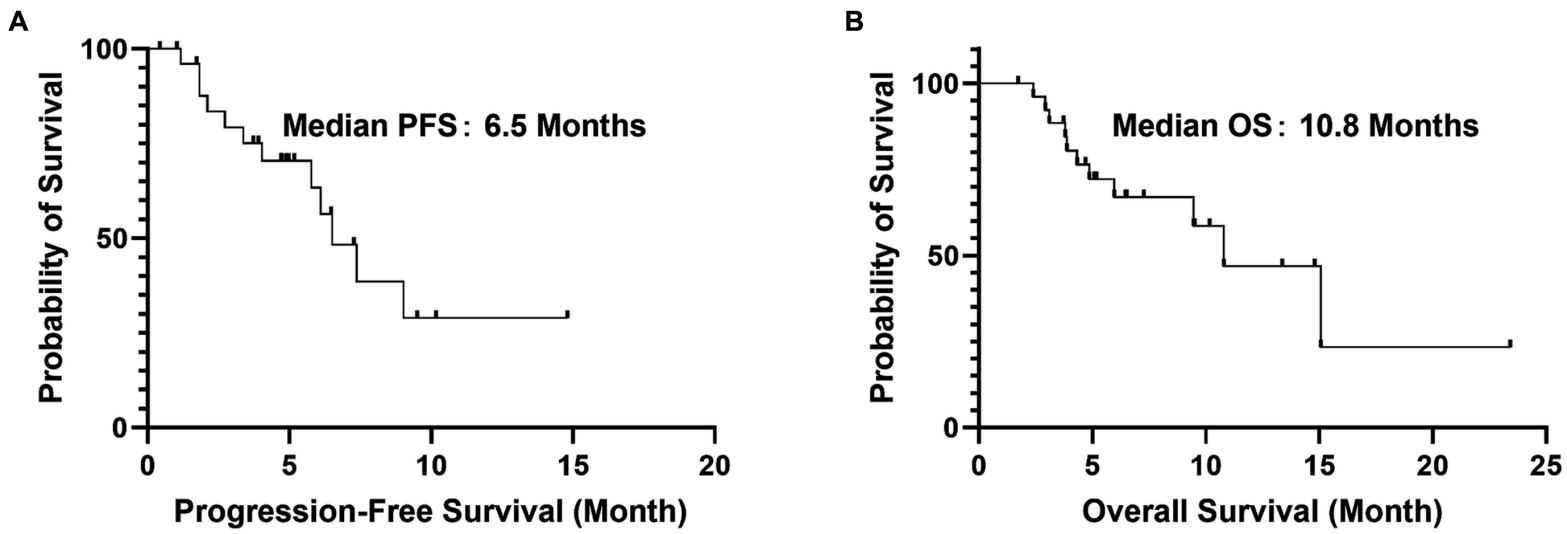
Kaplan–Meier curves stratified by prior anti-angiogenic therapy. **(A)** Progression-free survival (PFS). **(B)** Overall survival (OS). Patients without prior anti-angiogenic therapy (blue line, “No prior anti-angiogenic agents,” *n* = 21) had significantly longer PFS and OS compared to those with prior exposure (red line, “Prior anti-angiogenic agents,” *n* = 6). Median PFS: 9.1 months (no prior) vs. 3.0 months (prior); median OS: 13.5 months (no prior) vs. 5.7 months (prior). HR, hazard ratio; CI, confidence interval.

Stratified analysis revealed that prior exposure to anti-angiogenic therapy significantly influenced treatment outcomes. Patients who had not received prior anti-angiogenic agents (*n* = 21) exhibited markedly longer median PFS (9.1 vs. 3.0 months; HR = 0.29, 95% CI: 0.15–0.56; *p* < 0.001; Figure 2A) and median OS (13.5 vs. 5.7 months; HR = 0.42, 95% CI: 0.20–0.89; *p* = 0.025; Figure 2B) compared to those with prior exposure. In contrast, no significant differences in PFS or OS were observed between patients with G12C and non-G12C KRAS subtypes (G12C mPFS: 9.0 months vs. non-G12C: 6.0 months, *p* = 0.57; G12C mOS: 10.6 months vs. non-G12C: 9.0 months, *p* = 0.75).

### Safety

Treatment with sintilimab plus anlotinib was associated with a spectrum of adverse events consistent with the known safety profiles of each agent. All patients experienced at least one treatment-related adverse event (TRAE), and grade 3–4 TRAEs occurred in 25.9% (7/27) of patients, with hypertension (11.1%, 3/27) and hand-foot syndrome (7.4%, 2/27) being the most common (Table 2). No treatment-related deaths were reported.

**Table 2:** Treatment-related adverse events (*n* = 27).

Adverse event	All grades, *n* (%)	Grade 3–4, *n* (%)
Hypertension	16 (59.2)	3 (11.1)
Hand-foot syndrome	13 (48.1)	2 (7.4)
ALT increased	5 (18.5)	1 (3.7)
Proteinuria	7 (25.9)	1 (3.7)
Any TRAE	27 (100)	7 (25.9)

Adverse events were assessed and graded according to the National Cancer Institute Common Terminology Criteria for Adverse Events (NCI CTCAE), version 5.0. Data were retrospectively abstracted from medical records; under-reporting of mild or asymptomatic events is possible. ALT, alanine aminotransferase.

### Exploratory analyses

We evaluated the prognostic value of baseline inflammatory and nutritional indices, including the neutrophil-to-lymphocyte ratio (NLR) and platelet-to-lymphocyte ratio (PLR). However, in this cohort, neither a high NLR (cutoff = 2.99) nor a high PLR (cutoff = 176.33) was significantly associated with PFS (mPFS 6.0 vs. 9.0 months, *p* = 0.35; and 5.4 vs. 9.7 months, *p* = 0.28, respectively).

## Discussion

This multicenter retrospective study provides preliminary evidence that the combination of sintilimab and anlotinib may offer clinically relevant activity and an acceptable safety profile as a later-line therapy for patients with advanced *KRAS*-mutant NSCLC, warranting further prospective investigation. With a median PFS of 8.0 months and median OS of 12.4 months, this chemotherapy-free regimen represents a strategy worthy of future exploration, particularly in regions with limited access to *KRAS* G12C inhibitors. A key finding of our analysis is that patients without prior exposure to anti-angiogenic therapy had numerically longer median PFS (9.1 vs. 3.0 months) and OS (13.5 vs. 5.7 months) compared to those with prior exposure, suggesting a potential association between treatment history and clinical outcomes. This observation underscores the critical influence of prior therapy on treatment sequencing and clinical outcomes.

Our results compare favorably with existing later-line options for *KRAS*-mutant NSCLC. In the context of second-line therapy, the observed PFS of 8.0 months compares favorably with docetaxel (median PFS 3.0–4.2 months) and is within range of the PFS reported for sotorasib (5.6 months) and adagrasib (6.5 months) in KRAS G12C-mutant populations (10, 11). Moreover, the disease control rate of 92.6% is notable, particularly when considering that our cohort included both G12C (48.1%) and non-G12C (51.9%) subtypes (3). This suggests that the combination may provide clinical benefit across KRAS variants, addressing an important unmet need for non-G12C patients who are not eligible for G12C-specific inhibitors. The efficacy also aligns with that of the atezolizumab-bevacizumab-chemotherapy triplet (mPFS 8.1 months) from the IMpower150 cohort (12). The observed grade 3–4 toxicity rate of 25.9% in our cohort appears lower than the 60–70% reported in some prospective trials of chemo-immunotherapy combinations (13, 14); however, direct comparisons are limited by differences in data collection (retrospective vs. prospective) and potential under-ascertainment of adverse events in our study.

The association between prior anti-angiogenic therapy and inferior outcomes with the sintilimab-anlotinib regimen is logically consistent with expectations, as retreatment with agents targeting similar pathways may yield diminished benefit. We hypothesize that this may be driven by two interrelated mechanisms: first, prolonged anti-angiogenic pressure may induce a fibrotic, hypoxic tumor microenvironment characterized by impaired T-cell infiltration and function—a state often termed vascular “abnormalization” (4); second, tumor cells may undergo clonal selection under VEGF/VEGFR inhibition, leading to upregulation of alternative pro-angiogenic pathways (e.g., FGF, ANG-2) that diminish the efficacy of subsequent VEGFR inhibition (5). This biological rationale is consistent with subgroup analyses from the IMpower150 trial, wherein patients previously treated with bevacizumab derived less pronounced benefit from the atezolizumab-based combination (6).

When contextualized within the broader therapeutic landscape, our findings suggest that the sintilimab-anlotinib combination warrants further investigation. While its efficacy in our selected cohort appears numerically higher than historical data for anlotinib monotherapy (ALTER-0303: mPFS 4.8 months), direct cross-trial comparisons are limited by differences in patient selection, sample size, and study design (8). Regarding prior anti-angiogenic exposure, our findings differ from those of the ORIENT-31 study (9)—which evaluated sintilimab combinations in EGFR-mutant NSCLC after TKI failure—a distinct clinical and biological context. These differences highlight the need for prospective studies designed specifically for *KRAS*-mutant populations.

The safety profile observed was consistent with the known effects of each agent, and no new safety signals were identified. The most common grade 3–4 TRAEs were hypertension and hand-foot syndrome, which were readily managed with supportive care and dose modification. It is noteworthy that no grade ≥3 immune-related pneumonitis or hepatitis occurred in our cohort. While this contrasts with the higher rates (8–12%) sometimes observed with PD-1 inhibitor plus chemotherapy combinations (15), it is possible that the absence of chemotherapy, rather than a specific protective effect of VEGFR-TKI, contributed to this favorable safety profile.

Due to the modest sample size (*n* = 27) and limited number of events, multivariate analysis to adjust for potential confounders (e.g., age, ECOG performance status) was not feasible. Therefore, unmeasured confounding cannot be excluded, and the findings should be interpreted as hypothesis-generating rather than definitive. The lack of data on PD-L1 expression and tumor mutational burden also limits our ability to assess the contribution of these potential confounding factors. Additionally, the retrospective abstraction of safety data from medical records may have led to under-ascertainment of mild or asymptomatic adverse events. The inclusion criterion requiring completion of at least two treatment cycles may have introduced selection bias toward patients with better initial tolerance and longer intrinsic PFS, potentially overestimating the true treatment effect. These factors should be considered when interpreting the reported survival outcomes.

Future research should seek to prospectively validate “prior anti-angiogenic therapy” as a stratification factor in clinical trials. It will also be crucial to explore optimal therapeutic sequencing, such as prioritizing *KRAS* G12C inhibitors (e.g., fulzerasib) when available, followed by sintilimab-anlotinib upon progression. For patients with non-G12C mutations, investigating rational combinations such as VS-6766 plus defactinib (16) represents another promising direction.

## Conclusion

In summary, this case series provides hypothesis-generating evidence that the combination of sintilimab and anlotinib may have clinical activity in pretreated, advanced KRAS-mutant NSCLC. The observation of numerically longer survival in patients without prior anti-angiogenic therapy highlights the potential importance of treatment sequencing and warrants further investigation in prospective studies. In the context of constrained access to KRAS G12C inhibitors in some regions, this regimen represents a strategy deserving of future exploration.

## Data Availability

The original contributions presented in the study are included in the article/supplementary material, further inquiries can be directed to the corresponding author.
